# Molecular Characterization and Expression Changes of the *bcl2l13* Gene in Response to Hypoxia in *Megalobrama amblycephala*

**DOI:** 10.3390/cimb46020072

**Published:** 2024-01-25

**Authors:** Axin Zhang, Xuefei Guo, Kaikai Bao, Danyang Wu, Hong Liu, Zexia Gao, Huanling Wang

**Affiliations:** 1 Key Laboratory of Freshwater Animal Breeding, Key Laboratory of Agricultural Animal Genetics, Breeding and Reproduction, Ministry of Education, College of Fishery, Huazhong Agricultural University, Wuhan 430070, China; 15071318925@163.com (A.Z.); guoxf2016@hotmail.com (X.G.); 15207186192@163.com (K.B.); sgrwdy@163.com (D.W.); liuhong59@mail.hzau.edu.cn (H.L.); gaozx@mail.hzau.edu.cn (Z.G.); 2 Engineering Research Center of Green Development for Conventional Aquatic Biological Industry in the Yangtze River Economic Belt, Ministry of Education, Wuhan 430070, China

**Keywords:** *Megalobrama amblycephala*, *bcl2l13*, hypoxia, mitophagy, expression, subcellular localization

## Abstract

Hypoxia is a unique environmental stress, which not only reflects the insufficient oxygen supply of cells and tissues, but also occurs in various physiological and pathological environments. Mitophagy as a selective autophagy can recover and utilize damaged organelles and misfolded proteins to ensure normal cell functions and promote cell survival. Bcl2l13 (B-cell lymphoma-2 like 13) is reported to induce mitophagy as a functional mammalian homolog of Atg32. However, the function of the *bcl2l13* gene is still unclear in fish. Here the sequence and structure of the *bcl2l13* gene in *Megalobrama amblycephala* were identified and showed that *bcl2l13* contained an open reading frame (ORF) of 1458 bp for encoding 485 aa. Amino acid sequence analysis indicated that Bcl2l13, as a typical anti-apoptotic protein of the Bcl2 family, contained four BH domains, one BHNo domain, and one TM domain. Further study showed that Bcl2l13 was mainly located in the mitochondria, while its localization was changed within the whole cell after the TM domain was deleted. Real-time PCR analysis revealed that *bcl2l13* showed higher expression levels in early embryos. After hypoxia treatment, the mRNA levels of the *bcl2l13* and autophagy-related genes were significantly up-regulated in most detected tissues, and the *bcl2l13* transcription was regulated by Hif-1α mediated pathway. Additionally, the transcription activity of the *bcl2l13* promoter was further analyzed using luciferase reporter assays and showed the highest activity in the promoter region from −475 to +111. These results indicated that *bcl2l13* may play important roles in embryogenesis and hypoxia mediated autophagy in fish.

## 1. Introduction

Autophagy is a conserved biological metabolic process that refers to the encapsulation of cytoplasm and organelles into autophagosomes through bilayer membrane vesicles and transferring to vacuoles/lysosomes for decomposition and recycling [1]. Autophagy contains non-selective autophagy and selective autophagy. Among them, selective autophagy mainly includes cytoplasm-to-vacuole targeting (Cvt) pathway, mitochondrial autophagy, and peroxisome autophagy. Mitochondria-specific autophagy is also called mitophagy. Dysregulation of mitophagy is implicated in the development of chronic diseases including neurodegenerative diseases, metabolic diseases, and heart failure [2,3,4].

In yeast, the Atg32 protein is essential for mitophagy through its interaction with Atg8 and Atg11. Atg32 has a single transmembrane domain anchored to the outer mitochondrial membrane, and a WXXI motif for binding with Atg8 [5]. In humans (*Homo sapiens*), a mitophagy receptor, BCL2L13 (also known as Bcl-rambo), is identified [6] and can induce mitophagy in Atg32-deficient yeast, suggesting that BCL2L13 is a functional homolog of Atg32 [7,8]. Unlike some other BCL2 (B-cell lymphoma-2) like proteins that only have a BH3 domain, BCL2L13, as a member of the BCL-2 family contains four conserved N-terminal BH domains (BH1-4), one BHNo, and one transmembrane (TM) domains. The BH domains are involved in mitochondrial fragmentation, whereas the WXXI motif, a LC3 interacting region (LIR), is important for mitophagy [8]. The TM domain anchors BCL2L13 in the mitochondrial outer membrane [6]. Studies have confirmed that BCL2L13 functions in apoptosis, mitophagy, and mitochondrial fragmentation, and plays important roles in growth, development, and energy metabolism, while its dysregulation can lead to the pathological changes in some diseases [9].

Hypoxia is a major stressor that affects growth, development, reproduction, and even survival in aerobic species. In order to adapt to hypoxic environments, organisms evolutionarily generate diverse biological strategies, such as changes in protein synthesis, energy metabolism, mitochondrial respiration, etc. [10]. The best-studied mechanism of a response to hypoxia, is that hypoxia inducible factors (HIFs) activate the expression of a multitude of genes related with angiogenesis, glycolysis, apoptosis, proliferation, etc. [10]. Studies have confirmed that hypoxia promotes mitochondrial fission to induce mitophagy by BNIP3/BNIP3L and FUNDC1-mediated manners [11,12]. BNIP3 and BNIP3L are transcriptionally activated by HIF-1 in response to hypoxia [11]. BCL2L13 is found to be a mitophagy receptor located on the outer mitochondrial membrane and can mediate mitophagy through interaction with LC3 in a PRKN-independent manner [9], but its function in hypoxia response is still unclear.

Blunt snout bream (*Megalobrama amblycephala*) is a kind of herbivorous freshwater fish, that is distributed in the middle reaches of the Yangtze River. It has the characteristics of a wide feeding source, a fast growth rate and delicious fish meat [13]. Since the 1960s, *M. amblycephala* has been recognized as one of the most popular cultured species in the freshwater fish mixed-culture system in China. However, the tolerance to the hypoxic environment of *M. amblycephala* is weaker than other cyprinid fish, like *Carassius auratus*, which seriously affects the development of its production [14]. Hypoxia as one of the key environmental stressors can lead to mitophagy, but studies about the *bcl2l13* function in fish are not reported. In order to understand the potential functions of fish *bcl2l13* gene in the present study, we identified *M. amblycephala bcl2l13* and analyzed its expression patterns in different development stages and in adult tissues treated by hypoxia. Additionally, promoter activity and subcellular localization of *bcl2l13* were also analyzed. These results could help accelerate the understanding of *bcl2l13* function in fish, and provide a theoretical basis for exploring the role of *bcl2l13* in the mechanism of hypoxia response.

## 2. Materials and Methods

### 2.1. M. amblycephala Maintenance and Sample Collection

*M. amblycephala* samples (about 50 g) were obtained from an aquaculture breeding company in Hubei. The fish were fed twice per day with a commercial pellet diet (Haida, Guangdong, China) amounting to 3% of body weight in a 500 L temporary tank with a circulating water system (water temperature 25 ± 1 °C) for 2 weeks before the formal experiment. In the formal experiment, the fish were divided into 6 tanks of 100 L water (*n* = 10), of which 3 tanks were used as the control without any treatment, and the other 3 tanks were treated with hypoxia by filling nitrogen into the water. After maintaining the acute hypoxia (DO: 1.0 mg/L, T: 25 °C) for 0.5 h, 9 fish (3 fish per tank) in the hypoxia treatment group were anesthetized with 0.1% MS-222, blood samples were collected from the vena caudali, and then different tissues including the liver, spleen, muscle, brain, heart, and kidney were collected, quickly frozen in liquid nitrogen, and stored at −80 °C for total RNA extraction. Fish under normoxic conditions were used as the control. Same tissues from three fish (random selection, regardless of sex) from the same tank were mixed into one pool, and the three pooled samples were used to analyze gene expression. 

In order to investigate the spatial expression patterns of *M. amblycephala bcl2l13*, different tissues including liver, spleen, muscle, brain, heart, gill, intestine, kidney, ovary, and blood from adult individuals (*n* = 9) were collected under normoxic conditions. The embryos and larvae were obtained by artificial insemination from three pairs of parents. Embryos (at least 20 at every stage) were observed under an anatomic microscope and collected at the zygote stage, 2-cell stage, 8-cell stage, 32-cell stage, blastula stage, gastrula stage, neural plate stage, body segment appearance stage, eye vesicle stage, muscle functioning stage, hatching, and 4, 8, 10, 15 days post-fertilization (dpf). All samples were anesthetized with 0.1% MS-222, immediately frozen in liquid nitrogen, and stored at −80 °C for RNA extraction.

The experiments on fish in this study were approved by the Animal Ethics Committee of Huazhong Agricultural University (Wuhan, China, HZAUFI-2020-0022).

### 2.2. Real-Time PCR

Total RNAs from *M. amblycephala* embryos, larva, and different tissues were extracted using Trizol reagent (Invitrogen, Waltham, MA, USA). Genomic DNA was digested with RNase free DNase I (Invitrogen). A total RNA of 1 μg was reverse-transcripted to synthesize cDNA using M-MLV reverse transcriptase (Promega, Madison, WI, USA). Real-time PCR was performed in a 20 μL volume containing 1 μL cDNA, 0.4 μL primers (10 μM), and 10 μL 2 × Light Cycler 480 SYBR Green I PCR Master Mix (Roche, Indianapolis, IN, USA). The primers are shown in Table 1. The cycling parameters were 95 °C for 30 s, followed by 40 cycles of 95 °C for 5 s, 62 °C for 20 s, and 72 °C for 30 s. *β-actin* or *18S-rRNA* were used as the internal control [15]. All of the samples were analyzed in triplicate, and the expression of *bcl2l13* and autophagy related genes (*bnip3*, *beclin1*, *bax*, *parkin1*, *bnip3l*, *fundc1* and *p62*) were calculated as relative folds of the expression of the reference gene according to the ΔΔCt method [16].

### 2.3. Sequence Alignment and Analysis

The *bcl2l13* mRNA and genomic sequences were obtained from the GeneBank and the UCSC database (https://genome.ucsc.edu/) (accessed on 5 July 2018). Transcription factor binding sites were predicted on the 2 kb region upstream of the *bcl2l13* gene, using the JASPAR CORE Vertebrate database. The amino acids were deduced on ExPASy (http://www.expasy.ch/tools/dna.html) (accessed on 20 December 2020). Uniprot (https://www.uniprot.org/) (accessed on 20 December 2020) was used to analyze conserved domains. A multiple sequence alignment was conducted using ClustalW2 (http://www.ebi.ac.uk/Tools/msa/clustalw2/) (accessed on 21 December 2020). Phylogenetic tree analysis was performed by MEGA 7 with the neighbor-joining method [17].

### 2.4. Cell Culture and Plasmid Constructs

HeLa and FHM (Fathead minnow) cell lines (Cell Collection Centre for Freshwater Organisms, Huazhong Agricultural University) were maintained in a humidified atmosphere containing 5% CO_2_ in Dulbecco’s Modified Eagle Medium (DMEM) and M199 medium (Hyclone, Carlsbad, CA, USA), supplemented with 10% fetal bovine serum (FBS) and 1% penicillin-streptomycin at 37 °C and 28 °C, respectively. 

In order to analyze the effect of different domains of Bcl2l13 on subcellular localization, the cDNA sequences of *bcl2l13* with deleting different domains were amplified and cloned into the pEGFP-N1 plasmids (preserved in the laboratory) to express fusion proteins of the GFP-Bcl2l13 (pEGFP-Bcl2l13), GFP-Bcl2l13 with deleting TM domain (pEGFP-Bcl2l13-∆TM), GFP-Bcl2l13 with deleting BHNo domain (pEGFP-Bcl2l13-∆BHNo), or both BHNo and TM domains (pEGFP-Bcl2l13-∆BHNo-∆TM).

Genomic DNA was extracted from *M. amblycephala* embryos using a traditional phenol/chloroform DNA extraction method [18]. Different promoter fragments (−1608/+111, −998/+111, and −475/+111) located upstream of *bcl2l13* were amplified and directly inserted into the pGL3-basic vector, respectively, for promoter activity analysis. Additionally, in order to further analyze Hif-1α regulation to the *bcl2l13* transcription, three different promoter sequences, including three binding sites of the hypoxia response element, (HRE) were inserted into the pGL3-basic vector.

### 2.5. Cell Transfection, Luciferase Reporter Assays and Subcellular Localization

For luciferase reporter assays, HeLa cells were seeded in triplication to 24-well plates, and transiently co-transfected with 1 μg of the indicated reporter plasmids, 0.1 μg of pRL-TK plasmid (control) encoding Renilla luciferase or PCMV-myc-Hif1α, or PCMV-myc using Lipofectamine 2000 reagent (Invitrogen), according to the manufacturer’s instructions. At 48 h post-transfection, luciferase activities were measured using the dual-luciferase assay system (Promega) according to the manufacturer’s protocol.

For subcellular localization, FHM cells were transfected by pEGFP-N1, pEGFP-Bcl2l13, pEGFP-Bcl2l13-∆TM, pEGFP-Bcl2l13-∆BHNo, and pEGFP-Bcl2l13-∆BHNo-∆TM plasmids, respectively, using the Lipofectamine™ 2000 (Invitrogen), and stained by Mito Tracker Red CMXRos for mitochondria and DAPI for nuclei after 24 h of transfection. The subcellular localization of GFP fused Bcl2l13 was observed by a NIKON laser scanning confocal microscope with the NIS-Elements Viewer.

### 2.6. Statistical Analysis

All experimental data were expressed as mean ± standard error (mean ± SE), and each experiment was processed three times in parallel. SPSS Statistics 17.0 software was used for Student’s *t*-test or one-way analysis of variance (ANOVA). Duncan’s method was used for multiple comparisons of the experimental data to test the difference significance. The significance level was set as *p* < 0.05 and the extremely significant level was set as *p* < 0.01.

## 3. Results

### 3.1. Cloning and Molecular Characterization of M. amblycephala bcl2l13

**Figure 1:**
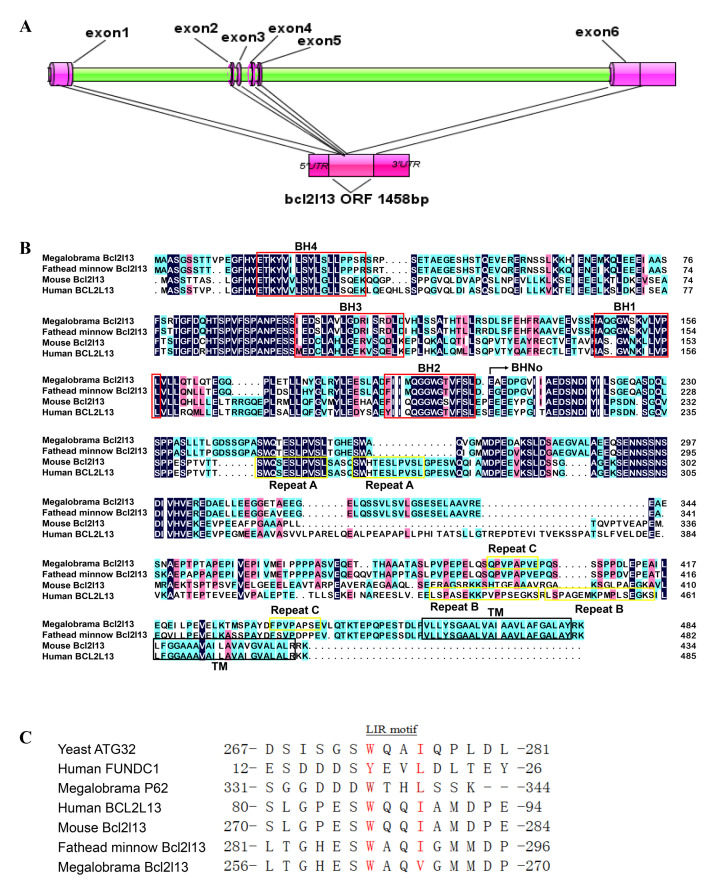
Sequence analysis of *M. amblycephala bcl2l13* gene. (**A**) Gene structure; (**B**) Comparison of the predicted amino acid sequences of *M. amblycephala* Bcl2l13 with the homologues of fathead minnow, mouse, and human. One short tandem repeat sequence (Repeat C) in *M. amblycephala* Bcl2l13 and two distinct short tandem repeats (Repeat A and B) in human BCL2L13 were indicated by boxes. Identical, similar, and low similar amino acids are shown in black, pink, and blue blocks, respectively. Repeat A–C, BH1-4, and transmembrane (TM) domains are shown in yellow, red, and black boxes, respectively. BHNo domains are indicated with an arrow; (**C**) and LC3 interacting region (LIR) motif (WXXL/WXXI/WXXV). The red part represents conserved amino acids within the LIR motif.

The 3179 bp *bcl2l13* cDNA was obtained, including an open reading frame (ORF) of 1458 bp, encoding 485 amino acids. Alignment between the cDNA and genome sequences showed that *M. amblycephala bcl2l13* was composed of six exons and five introns (Figure 1A). SMART domain analysis showed that Bcl2l13 characterized a C-terminal transmembrane (TM) domain (460–482 aa) anchored to the mitochondria and was a typical Bcl2 family anti-apoptotic protein with four BH domains. In addition, the BHNo domain (202–459 aa) and only one short tandem repeat sequence (Repeat C) was also found in *M. amblycephala* Bcl2l13, while there were two distinct short tandem repeats (Repeat A and B) in human BCL2L13 (Figure 1B). The WXXL/WXXI/WXXV motif for LC3 was found in BCL2L13 sequences from different species, the homologs (yeast ATG32), and human FUNDC1 (Figure 1C). 

**Figure 2:**
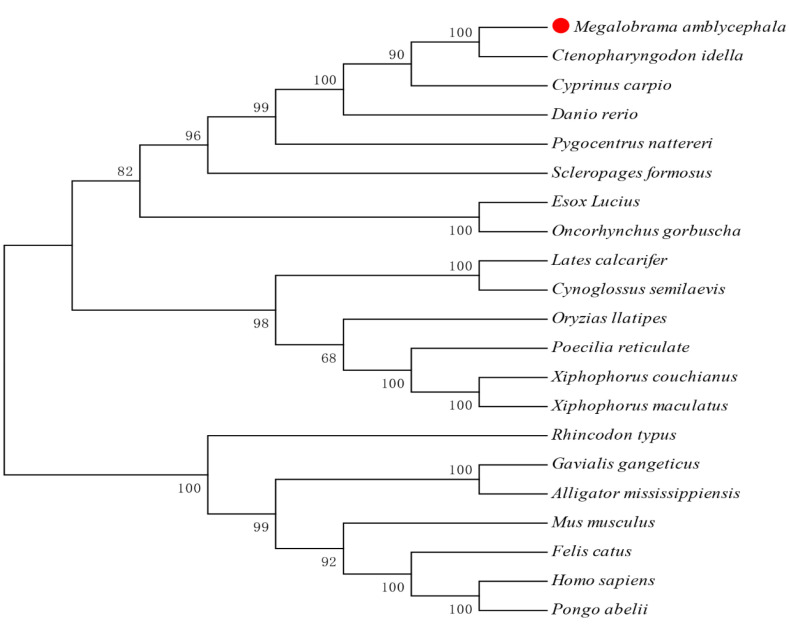
Phylogenetic tree analysis of Bcl2l13 from different species. The amino acid sequences are aligned and used to construct an evolutionary tree by MEGA7 using the neighbor-joining method. Red dot represents Bcl2l13 of *M. amblycephala* analyzed in this study.

Phylogenetic tree analysis showed that the amino acid sequences of Bcl2l13 were roughly divided into two branches. The aggregation of cartilaginous fish with mammals and reptiles was one large branch, and teleost fishes were clustered into the other separate large branch. *M. amblycephala* Bcl2l13 had the highest similarity with that of *Ctenopharyngodon idella*, which is similar to the species status in the evolutionary classification (Figure 2). 

### 3.2. Subcellular Localization of Bcl2l13

**Figure 3:**
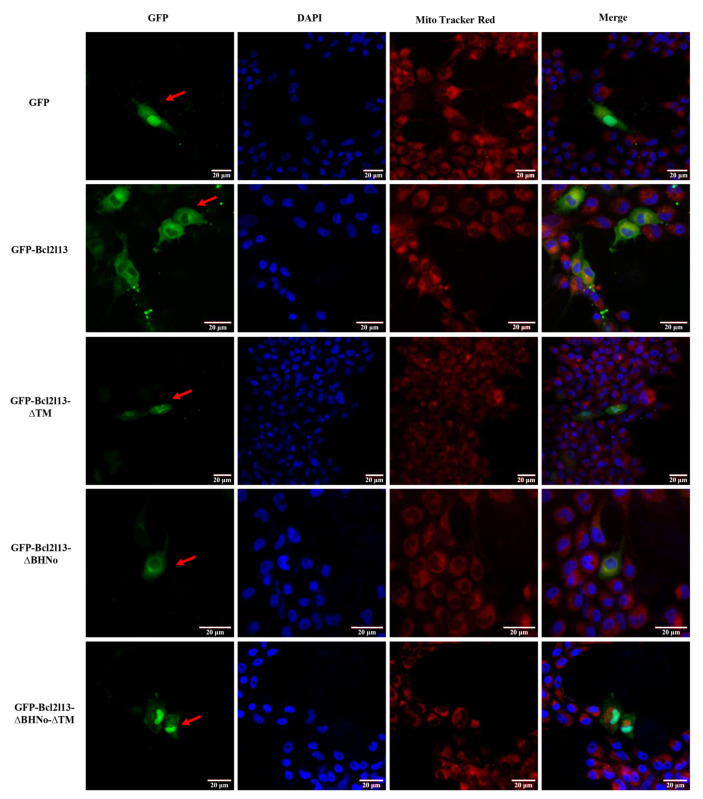
Subcellular localization of *M. amblycephala* Bcl2l13 protein. The plasmids expressing different fusion proteins were transfected into FHM cells, and fluorescence signals were observed at 24 h post transfection. GFP (control): pEGFP-N1 plasmid expressing GFP protein. GFP-Bcl2l13: the plasmid expressing GFP-fused Bcl2l13 protein. GFP-Bcl2l13-∆TM: the plasmid expressing GFP-fused Bcl2l13 protein lacking the TM domain. GFP-Bcl2l13-∆BHNo: the plasmid expressing GFP-fused Bcl2l13 protein lacking the BHNo domain. GFP-Bcl2l13-∆BHNo-∆TM: the plasmid expressing GFP-fused Bcl2l13 protein lacking the BHNo and TM domains. Red fluorescence marks mitochondria by the red fluorescent dye Mito tracker Red CMXRos, and blue fluorescence shows nuclei by DAPI dye. Red arrows represents cells expressing fusion protein or GFP. Scale size = 20 µm.

The protein localization in cells is important to understand its function. To confirm the subcellular distribution of *M. amblycephala* Bcl2l13, we constructed the vectors expressing GFP-fused Bcl2l13 with different domains, and transfected them into FHM cells. The results showed that green fluorescence was distributed throughout the cell in the control (pEGFP-N1), but mainly localized in the mitochondria after transfecting the plasmid (pEGFP-Bcl2l13), expressing GFP-fused Bcl2l13 protein. However, after the TM domain or both the BHNo and TM domains (pEGFP-Bcl2l13-∆TM and pEGFP-Bcl2l13-∆BHNo-∆TM plasmids) were deleted, Bcl2l13 was expressed throughout the cell, and while only the BHNo domain (pEGFP-Bcl2l13-∆BHNo plasmid) was lacked, Bcl2l13 localization was not changed (Figure 3). These results suggested that Bcl2l13 was localized in the mitochondria and the TM domain, not the BHNo domain, was necessary for Bcl2l13 localization.

### 3.3. Spatial-Temporal Expression Patterns of M. amblycephala bcl2l13

**Figure 4:**
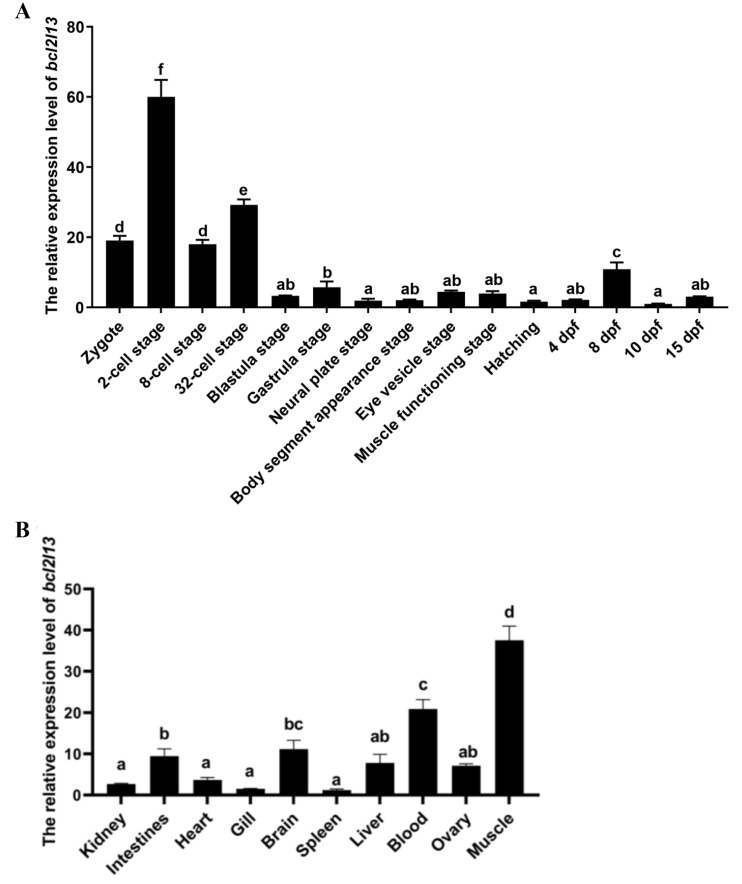
Expression patterns of *bcl2l13* during embryogenesis (**A**) and in different tissues (**B**) of *M. amblycephala. 18S-RNA* is used as the internal control during embryogenesis, and *β-actin* is used as the internal control in the different tissues. dpf: days post fertilization. The different letters on the black bars indicated a statistically significant difference at *p* < 0.05.

The expression pattern of *M. amblycephala bcl2l13* during embryonic development was detected by real-time PCR. The result showed that *bcl2l13* was dynamically expressed during embryogenesis, and the higher expression levels were shown before the 32-cell stage (Figure 4A).

In adult *M. amblycephala*, the *bcl2l13* gene was expressed in all of the detected tissues (kidney, intestines, heart, gill, brain, spleen, liver, blood, ovary, and muscle) under normoxic conditions, and the higher expression levels were found in the muscle, blood, brain and intestine (Figure 4B).

### 3.4. Hypoxia Effect on Expression of bcl2l13 and Autophagy-Related Genes

**Figure 5:**
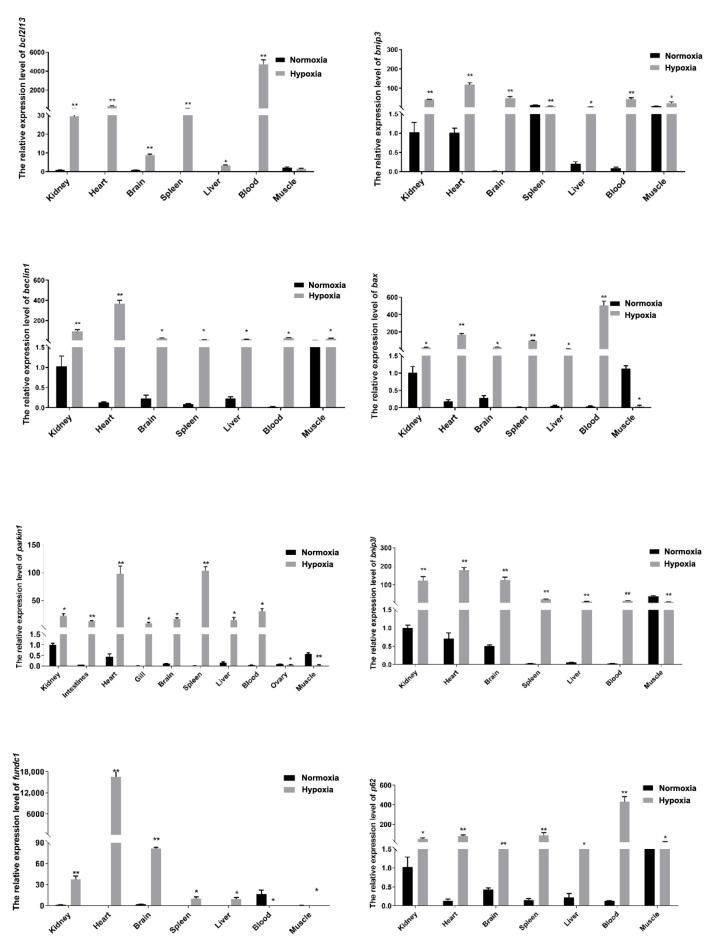
Hypoxia affects on expression of *bcl2l13* and autophagy related genes. Autophagy related genes including *bnip3*, *beclin1*, *bax*, *parkin1*, *bnip3l*, *fundc1* and *p62*. *β-actin* are used as the internal control. *Y*–axis values refer to the relative expression of target genes normalized by *β-actin,* based on the ΔΔCt method. Hypoxia, DO: 1 ± 0.2 mg/L. “*” stands for *p* < 0.05, “**” stands for *p* < 0.01.

In order to analyze hypoxia effects on the *bcl2l13* expression, the *bcl2l13* mRNA levels were detected after hypoxia treatment and the results showed that its expression was almost significantly increased in all of the detected tissues. Meanwhile, the expression of autophagy-related genes, *bnip3*, *bnip3l*, *beclin1*, *p62*, *bax*, *fundc1,* and *parkin1,* also showed significant up-regulation in most of the detected tissues after hypoxia treatment, especially in the kidney, heart, and brain (Figure 5). 

### 3.5. Activity Analysis of M. amblycephala bcl2l13 Promoter and Hif-1α Regulation

**Figure 6:**
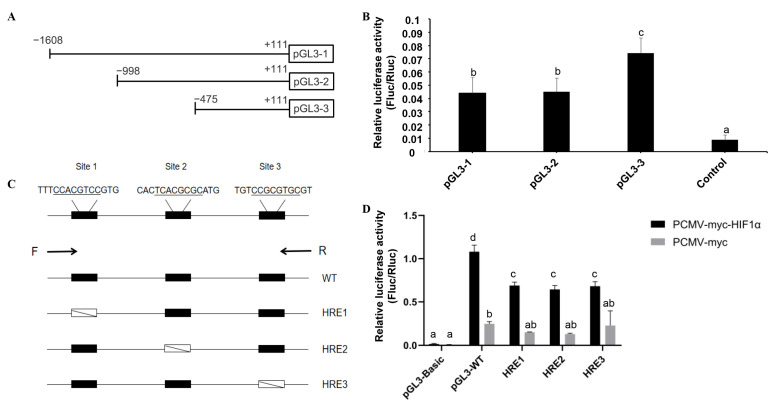
Promoter activity analysis of *M. amblycephala bcl2l13* and Hif-1α regulation. (**A**) Vectors containing different promoter regions were constructed, translation initiation was defined as +1. (**B**) Promoter activity analysis based on Dual Luciferase Reporter Assay System. The values were set relative to the control, Renilla luciferase activity. (**C**) The *bcl2l13* gene promoter and HRE (hypoxia response element, Hif-1α binding site) mutant region, including three HREs: HRE1: Site-directed mutagenesis of HRE1; HRE2: HRE2 site-directed mutagenesis; and HRE3: HRE3 site-directed mutagenesis. (**D**) Firefly luciferase activity was calibrated with Renilla luciferase activity. Data are reported as means ± SD and *n* = 3. The different letters on the black bars indicate statistically significant differences at *p* < 0.05.

To identify the core promoter region of *M. amblycephala bcl2l13*, three luciferase reporter vectors (pGL3-1, pGL3-2, pGL3-3) containing different 5′-flanking promoter regions were constructed (Figure 6A). The luciferase activities derived from the promoters with different sizes were determined using the Dual Luciferase^®^ Reporter Assay System. The luciferase activities of all the constructs were significantly increased compared to the pGL3-basic control (Figure 6B). As the data show in Figure 6B, the pGL3-3 promoter region (−475/+111) was able to drive the highest transcription level and showed a significant increase in luciferase activity compared with pGL3-1 (−1608/+111) and pGL3-2 (−998/+111), but there were no significant differences between pGL3-1 and pGL3-2. Analysis of transcription factor binding sites revealed that the −475 to +111 region contained potential binding sites of transcription factors Mef2C, Foxd3, Foxe1, Prdm1, Cdx2, Hoxc10, and Nkx6-3 (Table 2). And some potential transcription factor binding sites such as Myf6, Znf384, Nr3c1, Nr3c2, Grhl1 etc., were found especially in the region located from −998 to −475 (Table 2).

Additionally, we also found three Hif-1α binding sites in the *bcl2l13* promoter, and further luciferase activity assay confirmed that Hif-1α overexpression significantly increased luciferase activities (Figure 6C,D), indicating that the *bcl2l13* mRNA transcription was regulated by Hif-1α.

## 4. Discussion

BCL2L13 is a BCL2-like protein that plays an important role in promoting apoptotic activity and mitophagy, regulating many physiological processes such as growth, development, and energy metabolism [9]. Currently, most studies mainly focus on humans, mice, and yeast, but the *bcl2l13* function is still unclear in fish. Therefore, in the present study, the economic fish *M. amblycephala* was taken as the research object to study the potential function of this gene.

Our results showed that the predicted protein of *M. amblycephala bcl2l13* contained a transmembrane domain (TM), four BH domains, and a putative LIR motif, WAQV. Cellular localization showed that *M. amblycephala* Bcl2l13 was mainly localized in the mitochondria, but its localization in cells was changed when the TM, not BHNo domain, was deleted, indicating that TM domain is crucial to its localization. BCL2L13 of humans and mice also have a TM domain located in the outer membrane of the mitochondria, and the conserved LIR sequence for binding LC3, which is crucial for mitophagy [9,10]. Removal of the C-terminal membrane anchor of human BCL2L13 not only resulted in the loss of mitochondrial localization but also induced the cell death activity [10]. Mitophagy or apoptosis was not induced as the TM region was mutated or knocked out [9]. Unlike anti-apoptotic proteins in the BCL2 subfamily, BCL2L13 overexpression can promote apoptosis, and further study found that two distinct short tandem repeats in the C-terminal instead of the BH domains can induce cell apoptosis [10]. The only one distinct short tandem repeat was found in *M. amblycephala* Bcl2l13, but whether the BHNo domain could induce cell apoptosis needed further research. 

The *BCL2L13* mRNA was expressed in human oocytes and performed pro-apoptotic activity to help embryonic development [8,19]. During embryo development, *M. amblycephala bcl2l13* was highly expressed before the 32-cell stage, and then significantly decreased, which was similar to the expression pattern of *Bcl2l13* in the rhesus monkey [20,21]. Under normoxic conditions, a higher expression of *bcl2l13* was detected in the muscle, brain, blood, and intestine of *M. amblycephala*, suggesting that *bcl2l13* high expression was probably associated with more abundant mitochondria in these tissues. *BCL2L13* was highly expressed in skeletal muscle to raise physical endurance in indigenous groups from Northern Mexico [22]. Additionally, exercise training also increased BCL2L13 content in skeletal muscle, which is related with mitochondrial content and muscular efficiency for ATP supply [9,23]. High expression of *BCL2L13* was also an independent prognostic factor for childhood acute lymphoblastic leukemia [24,25]. Collectively, these results suggest that *bcl2l13* plays important roles in various tissues and early development of *M. amblycephala*. 

In order to investigate the effect of hypoxia on the *bcl2l13* expression, real-time PCR was performed and showed that the expression increased significantly in the kidney, heart, brain, spleen, liver, and blood after hypoxia treatment. Luciferase activity assay further confirmed that *bcl2l13* was probably regulated by the Hif-1α mediated pathway after hypoxia treatment. The mRNA expression of *BCL2L13* was dramatically increased in hypoxic cardiomyocytes and involved in hypoxia-induced oxidative stress and cell apoptosis [26]. In oxygen glucose deprivation/reoxygenation (OGD/R)-treated Neuro-2a (N2A) cells, *BCL2L13* mRNA expression was enhanced by miR-98-5p, leading to aggressive apoptosis, ER stress, inflammation, and oxidative stress [27]. Some studies have found that hypoxia can increase the Hif-1α expression, and induce autophagy by BNIP3/BNIP3L and P62 with binding to the LC3, one of the most classic mitophagy markers [15,28,29]. Interestingly, the expression pattern of *M. amblycephala bcl2l13* after hypoxia treatment was similar to some autophagy-related genes, such as *bnip3*, *bnip3l*, *beclin1*, *p62*, *bax*, *fundc1* and *parkin1*, and Bcl2l13 was localized by the mitochondria and contained LC3 interacting region (LIR) motif. These results suggested that *bcl2l13* may be involved in hypoxia induced mitophagy via LIR-mediated interactions.

The cloned 5′-upstream regions of *M. amblycephala bcl2l13* showed higher promoter activity after being transfected into FHM cells, compared with the control. Here, luciferase activity was significantly up-regulated after deletion of the −998 to −475 region, and we speculated that the basic regulatory elements required for *bcl2l13* promoter activity might be located in the −475 to +111 region, and the −998 to −475 region had negative regulatory factors. Additionally, transcription binding sites of Myf6 and Mef2 were found in the promoter of *bcl2l13*; Myf6 and Mef2 have been identified to play key regulation roles in muscle growth and development [30,31]. Our study found that *bcl2l13* had the highest expression in muscle tissue, suggesting that *bcl2l13* probably plays an important function in muscle development by Myf6 and Mef2 regulation.

In summary, the present study cloned the cDNA of *M. amblycephala bcl2l13*, analyzed its promoter activities, and characterized its expression patterns. Bcl2l13 was found to be mainly localized in the mitochondria, and the TM domain, not the BHNo domain, played a decisive role in the localization. The *bcl2l13* gene may be involved in hypoxia induced mitophagy in *M. amblycephala*. These results indicate that *bcl2l13* plays important roles in embryonic development and hypoxia response, which will contribute to further understanding regulation mechanisms of hypoxia response.

## Figures and Tables

**Table 1:** Primers used in the experiments.

Name of Primers	Sequence of Primers (5′-3′)	Use
pro-bcl2l13-F-1	CCTTGCAAAGATGGACAGACT	promoter
pro-bcl2l13-F-2	CGTGGCTTCATCCTCTTG	
pro-bcl2l13-F-3	CTCATATCAAGCAGTCAAGCG	
pro-bcl2l13-R	ATCTCTTACCCTCCGCTGTC	
bcl2l13-WT-F	CGACGCGTCTTACACCATTTGAGGGCAG	HRE confirmation
bcl2l13-WT-R	GGAAGATCTTTACAGTTTTCACAAGTCCT	
bcl2l13-WT-HRE1-Fm	CGTTTAAGATGAAGTGTGCGT	
bcl2l13-WT-HRE1-Rm	ACGCACACTTCATCTTAAACG	
bcl2l13-WT-HRE2-Fm	TCCCCCGGGATGTCTGAACAC	
bcl2l13-WT-HRE2-Rm	TCCCCCGGGGAGTGGTGTCCATG	
bcl2l13-WT-HRE3-Fm	TCCCCCGGGGTTACATTGTAC	
bcl2l13-WT-HRE3-Rm	TCCCCCGGGGGACACCCACTGCAC	
bcl2l13-ORF-F	CGAAGCTTATGGCTGCCTCTGGCTCCTCCACCACAGTG	ORF
bcl2l13-ORF-R	GCGGATCCCTCTTCTTCCTGTAGGCCAGC	
bcl2l13-qRT-F	TGGGCAGCGAAAGCGAACT	Real-time PCR
bcl2l13-qRT-R	ACGGGCAATGAGGCGGTAG	
bnip3l-qRT-F	GAGGAGGATGATGGGATGGT	
bnip3l-qRT-R	AGTTACTACTACGGCTGGATTCG	
bnip3-qRT-F	CGTTCCAGCACACTCAGCAT	
bnip3-qRT-R	AGTAGTAATACGCCTTCCGA	
bax-qRT-F	CTTTTCTACTTTGCGTGCCG	
bax-qRT-R	CTGCCAGGAAAACCCCAA	
becn1-qRT-F	GAGTTGCCATTGTATTGC	
becn1-qRT-R	GAACCTCCACTGCCACCG	
parkin-qRT-F	GCTGTGGGTTTGTGTTTT	
parkin-qRT-R	ATGAGTGGTTTTGGCTAT	
fundc1-qRT-F	AGGATGGTGTGCTGGATA	
fundc1-qRT-R	CAGGGGCTGCTTTGTTTG	
p62-qRT-F	GCAGTGATGAGGAATGGA	
p62-qRT-R	GGACCCTGTGTGTCGCTTGT	
18S-rRNA-qRT-F	CGGAGGTTCGAAGACGATCA	
18S-rRNA-qRT-R	GGGTCGGCATCGTTTACG	
β-actin-qRT-F	ACCCACACCGTGCCCATCTA	
β-actin-qRT-R	CGGACAATTTCTCTTTCGGCTG	

**Table 2:** Potential transcription factor binding sites in the −475 to +111 and −998 to −475 regions.

Name	Score	Predicted Sequences	Region
MA0497.1.MEF2C	15.95222	GAAACAAAAATAGAT	−475 to +111
MA0041.2.FOXD3	14.70476	AGAACAAACAAACAAA	
MA1487.1.FOXE1	14.64539	CCTAGAACAAACAAA	
MA0508.2.PRDM1	13.98241	TCACTTTCAA	
MA0042.1.FOXI1	13.57833	TTTTGTTTGTTT	
MA0465.2.CDX2	13.49907	TTGCAATAAACG	
MA0905.1.HOXC10	13.41888	GTCATTAAAT	
MA1530.1.NKX6-3	13.38798	GTCATTAAA	
MA0869.2.Sox11	13.35816	GAGCACAAAGGA	
MA0514.1.Sox3	13.29434	CCTTTGTGCT	
MA0909.3.Hoxd13	13.01262	TGCAATAAAC	
MA0667.1.MYF6	16.66626	TGTACACAATGTTCT	−998 to −475
MA1125.1.ZNF384	16.30834	TTGTACACAATGTTCTA	
MA0113.2.NR3C1	16.2096	ACCACTTAA	
MA0727.1.NR3C2	15.97045	ATTTATTTTTTT	
MA0647.1.GRHL1	14.76465	CGCACGCGGA	
MA1640.1.MEIS2	14.7477	GTACAATGTAA	
MA0124.2.Nkx3-1	14.69338	TTGTACACAAT	
MA1113.2.PBX2	14.62967	AACATTGTG	
MA1968.1.TFCP2	14.54535	TAAATCACTT	
MA0007.2.AR	14.41048	ACACAATGTTC	
MA0122.3.Nkx3-2	14.16071	TTAAGTGGT	
MA1511.1.KLF10	14.00595	CGCGTG	
MA0672.1.NKX2-3	13.95893	CGCGTG	
MA0122.2.NKX3-2	13.94363	CGCGTG	
MA1963.1.SATB1	13.54465	CACCTT	
MA0632.2.TCFL5	13.49938	CACCTT	
MA0506.1.NRF1	13.23179	ATCAAA	
MA1639.1.MEIS1	13.08374	TTAAGTG	
MA0624.2.Nfatc1	13.01802	ATGAGTGGC	

## Data Availability

Data are contained within the article.
